# Acute Cardiorenal Syndrome Type 1 in Patients With Congestive Heart Failure Exacerbations Is Not an Indicator of Poor Outcome and Increased Mortality

**DOI:** 10.14740/cr332w

**Published:** 2014-05-15

**Authors:** Eric Arguelles, Carolina de Elia, Zoran Lasic

**Affiliations:** a Department of Internal Medicine, Jamaica Hospital Medical Center, Jamaica, NY, USA; b Department of Internal Medicine, New York Methodist Hospital, Brooklyn, NY, USA; c Department of Cardiology and Interventional Cardiology, Jamaica Hospital Medical Center, Jamaica, NY, USA

**Keywords:** Cardio-renal syndrome, Heart failure, Prognosis

## Abstract

**Background:**

Over one million patients are hospitalized each year with acute decompensated heart failure (ADHF) in the US. Approximately 20% to 40% of them will develop acute cardiorenal syndrome type 1 (ACRS1) via multiple mechanisms.

**Methods:**

From January 2010 to December 2010, 197 patients were diagnosed with ADHF. Initial N-terminal pro-brain natriuretic peptide (NT-pro BNP), creatinine levels throughout hospital stay, use of invasive mechanical ventilation, length of hospital stay and death were assessed for each patient. ACRS1 was diagnosed when an increase of creatinine > 0.3 mg/dL from baseline was noted during hospital stay. We sought to investigate whether presence of ACRS1 is associated with increased length of stay, need for mechanical ventilation and increased in-hospital mortality in patients admitted with ADHF.

**Results:**

Total of 61 (31%) patients experienced ACRS1. Mean hospital stay for ACRS1 patients was 8.43 ± 6.28 days while for non-ACRS1 was 5.06 ± 4.19 (P < 0.0001). Twenty-eight (14%) patients required invasive mechanical ventilation (11 non-ACRS1 vs. 17 ACRS1). ACRS1 was associated with more frequent use of invasive ventilation (odd ratio 3.45, CI 1.52 - 7.79, P = 0.003). Fifteen (8%) patients expired (8 non-ACRS1 vs. 7 ACRS1). There was no difference in mortality between groups (odd ratio 2.07, CI 0.72 - 6.00, P = 0.18).

**Conclusions:**

Development of ACRS1 was not associated with increased incidence of in-hospital mortality, but it prolonged hospital stay and need for mechanical ventilation.

## Introduction

Over one million patients are hospitalized each year with acute decompensated heart failure (ADHF) in the US. Approximately 20% to 40% of them will present with acute kidney injury (AKI) as defined by an abrupt, within 48 h, absolute increase in the serum creatinine concentration of ≥ 0.3 mg/dL from baseline or an increase in the serum creatinine concentration of ≥ 50 percent as proposed by the Acute Kidney Injury Network (AKIN). These criteria have been extensively and repeatedly revised deriving in subsequent classification of the syndrome according to the extent of the kidney function change where each stage is directly related to the degree of severity and prognosis [1-7]. AKI as a complication of acute heart failure (AHF) and/or acute coronary syndrome is generally known as acute cardiorenal syndrome type 1 (ACRS1) which often occurs early after presentation to the hospital can be due to either the AHF physiopathology itself or to intense loop diuretic treatment [8]. These patients are prone to higher morbidity and mortality indices [9, 10]. Our study focuses on the role of ACRS1 as a predictor of outcome and mortality on AHF patients.

## Methods

This study was conducted at Jamaica Hospital Medical Center, Jamaica, New York, USA. It is a retrospective cohort study on 372 patients admitted and discharged from January 1, 2010 to December 31, 2010 with a primary or secondary admission diagnosis of AHF/congestive heart failure (CHF) exacerbation. After revising the admission diagnosis of all cases, we decided to include in our study only those patients where the primary diagnosis was AHF/CHF exacerbation or when the primary diagnosis could be a presentation of, or be directly related to the aforementioned primary diagnosis, for example, acute respiratory failure, acute on chronic respiratory failure, myocardial infarction, atrial fibrillation or flutter, hypertensive heart disease with heart failure, cardiomyopathy, and so on. We excluded those patients that met the following criteria: primary admission diagnosis unlikely related to AHF/CHF exacerbation: for example, hip fracture, upper extremity deep vein thrombosis, septicemia, intracerebral hemorrhage, uncontrolled diabetes mellitus, and so on, 30 patients were excluded; no available N-terminal pro-brain natriuretic peptide (NT-pro BNP) on admission or at least 2 weeks before (56 patients) and/or an initial NT-pro BNP less than the cut-off point for AHF/CHF exacerbation according to the current laboratory assay used during that period of time (see below, 9 patients); any disposition other than discharged or deceased, for example, transferred to other hospital (36 patients), signed out against medical advice (18 patients); insufficient laboratory data to trend creatinine levels upon and/or over admission, namely less than two basic metabolic profile (BMP) during admission (54 patients) and no echocardiogram obtained during or at least 6 months before or after discharge (22 patients). The final number of patients included in the study was 197. The study protocol was approved by the Institutional Ethics Committee.

We review a set of clinical and laboratory data including the following variables: initial serum NT-pro BNP levels, creatinine levels upon admission and throughout hospital stay, use of invasive mechanical ventilation, occurrence of shock during hospital stay and/or death and length of hospital stay. Quantitative measurement of NT-pro BNP was performed using the VITROS ECi/ECiQ Immunodiagnostic Systems (REF 6802156) to aid in the diagnosis of CHF. Cut off values to exclude AHF/CHF exacerbation were less than 125 pg/mL for patients < 75 years old and < 450 pg/mL for those ≥ 75 years old [11-17]. Values upon admission or at least 2 weeks before were logged as the initial values. Quantitative measurement of creatinine levels was undertaken using the VITROS CREA Slide method (REF 680 2584) with normal values for serum creatinine in males of 0.7 - 1.3 mg/dL and for females of 0.6 - 1.0 mg/dL according to results from an internal study of apparently healthy adults from a working population (serum: 113 males and 195 females) where these serum reference intervals represented the central 95%. The lowest and highest values during admission as well as those upon admission were logged and used to calculate maximal creatinine increase when applicable. Patients were ultimately classified as having experienced ACRS1 or not during admission.

### Statistical methods

The primary end point was the impact of ACRS1 on hospital stay length. Second end points were relationship of ACRS1 with mortality and morbidity as reflected in incidence of shock and/or use of invasive mechanical ventilation. All data were numerically coded and continuous variables were expressed as mean values and standard deviations while categorical variables were expressed as percent. Differences in prognostic and incidence of complications between the two groups (ACRS1 positive/negative) were analyzed using percentages, independent sample *t*-tests for means comparisons and odd ratios for non-numerical variables associations with a 95% confidence interval and a P < 0.05 level. All analyses were conducted using IBM SPSS statistics v. 18 (Chicago, IL).

## Results

From the total of 197 patients included in our study, 61 (31%) experienced ACRS1 anytime during the hospital stay according to the above mentioned criteria. Mean hospital stay in days and standard deviation for non-ACRS1 were 5.06 and 4.19 respectively, while for the ACRS1 groups were 8.43 and 6.28 respectively. There was a statistically significant difference at 95% confidence interval between the two means (P = 0.000017) supporting the association between ACRS1 incidence and longer hospital stay. ACRS1 was significantly associated with the use of invasive ventilation (odd ratio 3.4456, 95% CI 1.5231 - 7.7948, P = 0.0030). In terms of morbidity and mortality, only five (2%) patients experienced shock during the hospital stay while 15 (8%) expired. There was no statistically significant association between these two variables and the incidence of ACRS1 (odds ratio 3.4655, 95% CI 0.5640 - 21.2938, P = 0.1797 and odds ratio 2.0741, 95% CI 0.7164 - 6.0051, P = 0.1786, respectively).

## Discussion

Acute cardiorenal syndrome (ACRS) occurred in our sample population into the range of the revised literature [1-3]. ACRS1 on the other hand was found to be associated with longer hospital stay and increase use of invasive mechanical ventilation in our research although no relationship was found with increased incidence of shock or death. The results are partially concordant with those from other authors that found a positive association not only with longer hospital stays and increase use of invasive mechanical ventilation but with more frequent complications, including shock and even death [18-20].

### Study limitations

The study population consisted of 197 patients; the sample size was limited and originated from a single center. A large population from several facilities could be used in order to increase the generalizability of the findings.

### Conclusions

As in our study, ACRS1 was not consistently related with more frequent complications and/or death as evidenced in the reviewed literature. Nevertheless, its incidence did translate into longer hospital stay and need for more frequent invasive ventilation techniques. It continues to be a continuous topic of debate and ongoing research where prompt recognition and timely management could represent shorter hospital stays and better outcome.
